# Three-Dimensional In Vitro Tumor Spheroid Models for Evaluation of Anticancer Therapy: Recent Updates

**DOI:** 10.3390/cancers15194846

**Published:** 2023-10-04

**Authors:** Pallavi Nayak, Valeria Bentivoglio, Michela Varani, Alberto Signore

**Affiliations:** Nuclear Medicine Unit, University Hospital Sant’Andrea, Department of Medical-Surgical Sciences and of Translational Medicine, Faculty of Medicine and Psychology, “Sapienza” University of Rome, 00189 Roma, Italy; valeria.bentivoglio@uniroma1.it (V.B.); michela.varani@uniroma1.it (M.V.); alberto.signore@uniroma1.it (A.S.)

**Keywords:** 3D spheroids, cancer therapy, micro-environment, nanotechnology, nuclear medicine, theragnostics

## Abstract

**Simple Summary:**

Cancer is a global public health issue. The development and use of in vitro cellular models with pre-clinical animal models are essential to elucidate the complex biology of cancer and test new diagnostic and therapeutic options. Three-dimensional (3D) tumor models are particularly important as they can accurately mimic the behavior of solid tumors. This review article critically discusses the suitability of 3D spheroid models in oncological research.

**Abstract:**

Advanced tissue engineering processes and regenerative medicine provide modern strategies for fabricating 3D spheroids. Several different 3D cancer models are being developed to study a variety of cancers. Three-dimensional spheroids can correctly replicate some features of solid tumors (such as the secretion of soluble mediators, drug resistance mechanisms, gene expression patterns and physiological responses) better than 2D cell cultures or animal models. Tumor spheroids are also helpful for precisely reproducing the three-dimensional organization and microenvironmental factors of tumors. Because of these unique properties, the potential of 3D cell aggregates has been emphasized, and they have been utilized in in vitro models for the detection of novel anticancer drugs. This review discusses applications of 3D spheroid models in nuclear medicine for diagnosis and therapy, immunotherapy, and stem cell and photodynamic therapy and also discusses the establishment of the anticancer activity of nanocarriers.

## 1. Background

Cancer is a multifactorial disease caused by abnormal cell behaviors, such as sustained proliferative molecular networks (e.g., cell death resistance, angiogenesis, drug resistance) and immune evasion properties that lead to cell replication and invasion/migration characteristics associated with metastases [1]. Drug discovery research faces significant challenges in developing new drugs and determining their pharmacokinetics in cancer patients [2,3,4]. Since 1940, clinical treatment techniques for cancer treatment, such as chemotherapy, surgery, and radiotherapy, have been the most widely used. On the other hand, chemotherapeutic drugs are frequently non-specific, have a low concentration at the tumor site, and are rapidly eliminated from the blood. In addition, cancer cells can develop resistance to anticancer drugs at the beginning of treatment or after repeated administrations. As a result, cancer cells can escape the drug’s activity, resulting in low therapeutic efficacy. To overcome the resistance of solid tumors, the scientific community and the pharmaceutical industry have invested in developing highly effective anticancer treatments. New compounds must adhere to the standards set by regulatory organizations like the Food and Drug Administration (FDA) and the European Medicine Agency (EMA) before being used in the clinic. All anticancer therapeutics must be tested in vitro, in vivo, and in humans. The most common in vitro technique for such screenings is two-dimensional (2D) cell cultures, which Harrison first established in the early 19th century. This method is simple, reproducible, and affordable. However, in vivo, solid tumor characteristics and treatment resistance cannot be simulated in flat 2D cell culture models. Consequently, many ineffective drugs might proceed to in vivo testing, contributing to the abuse of animals in research and extending and increasing the cost of the drug development process. In light of these considerations, new and improved in vitro models have been studied to enhance the identification of therapeutic alternatives during the early phases of drug development and, consequently, to reduce the usage of laboratory animals. These models can replicate the characteristics of human tumors more closely than 2D models and often even better than animal models [5,6].

## 2. Three-Dimensional Models: An Alternative to In Vivo Models

Pre-clinical research primarily relies on 2D cell cultures and animal models, which is still a key experimental strategy in translational cancer research. Cell culture techniques are accessible, affordable, and well-suited for high-throughput toxicity and drug screening tests. Two-dimensional cell cultures, on the other hand, are simplistic representations of tumors that do not capture all of the fundamental cellular architecture and interactions that occur in vivo.

Patient-derived tumor xenografts (PDX) have a cellular complexity similar to the tumor and can maintain tumor heterogeneity. PDTXs are frequently implanted in immunocompromised deficient hosts, necessitating the regeneration of autologous immune cells to study immunity. Major drawbacks of PDXs are their poor implantation rates, long immune reconstitution processes, high cost, and extended generation times for humanized models [5]. 

Conversely, cancer is a highly heterogeneous illness with a complex and dynamic tumor microenvironment (TME). Cellular (stem cells, tumor epithelium, fibroblast, endothelial cells) and non-cellular (cytokines, chemokines, extracellular matrix (ECM), and growth factors) components of TME significantly impact tumor development, potentially influencing therapeutic development outcomes. As a result, pre-clinical models that mimic tumor biology in vivo are crucial for accurately assessing therapeutic toxicity and efficacy [6]. Hence, three-dimensional (3D) cell cultures have been designed as an alternative to 2D tissue cultures, as they better mimic and replicate the architecture of in vivo tissue. Table 1 depicts the comparison of different features of culture models. The 3D architecture of human solid tumors, which offers ideal conditions for cellular organization, proliferation, and differentiation, is one of their key characteristics. 

In light of this, in vitro 3D cell culture approaches (both scaffold-based and scaffold-free) have developed in recent years as a workable alternative to in vivo animal testing for the aim of drug screening (Table 2) [7,8,9,10]. 

To replicate microenvironment properties in a 3D model, the scientific community developed many platforms to address various levels of complexity, such as cells seeded on pre-formed porous scaffolds/fibrous materials or encapsulated in biomaterials made of water-soluble polymers called hydrogels. In addition, tissue physiology can be replicated using adult or pluripotent stem-cell-derived organoids, a self-organized 3D tissue culture created from stem cells to reproduce a portion or the majority of an organ’s complexity. In tissue engineering, scaffold-based strategies for growing cells in a 3D environment are highly prevalent [11].

Cells can also be cultured in a multi-channel 3D microfluidic cell culture device that mimics the mechanics, activities, and physiological responses of specific organs or systems [12]. Scaffold-free 3D cell cultures encompass all techniques that promote cell development without using external artificial platforms. These approaches stimulate the production of 3D microtissues as spheroids or multicellular tumor spheroids, in which cells build their own ECM [13].

## 3. The Need for 3D Spheroid Models

**Figure 1:**
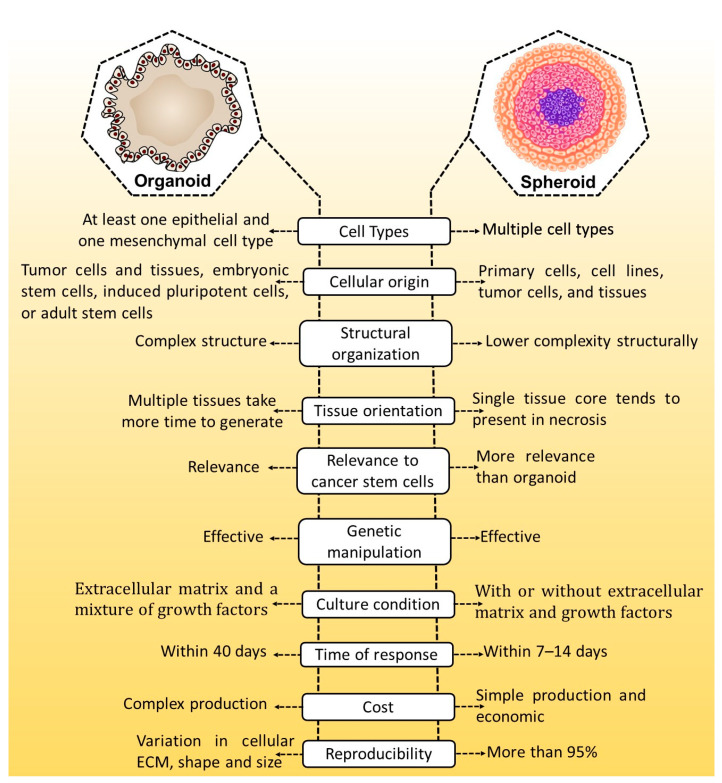
A comparison of organoid with spheroids models.

Three-dimensional cell cultures can be used as a substitute for laboratory organisms, reducing the need for animal testing, and toxicity levels for specific 3D cell cultures are similar to those in animal studies [14,15,16]. Various 3D models, such as spheroids, tissue explants, and organoids, have been developed [17]. Spheroids, normally cultured as free-floating aggregates of spherical cellular units, are considered to have a low level of complexity in mimicking the structure of tumors. Additionally, cellular spheroids have distinct cell phenotypes that match the structure of actual tumors, such as necrotic, proliferating, and non-proliferating cells [18]. The earliest evidence of cellular spheroid formation was discovered in 1944 by Holtfreter, while in 1970, Sutherland and coworkers developed a methodology for culturing spheroid cells [19,20]. Several strategies for creating cellular spheroids have been established thus far. The primary condition for forming cellular spheroids is that cell-to-cell adhesion must be higher than cells to substrates [21]. Organoids are 3D-grown cells with structural units that resemble the structure and function of organs in vivo [22]. Three-dimensional cultures can be established with or without the support of an ECM scaffold. Organoid and spheroids culture models serve distinct and complementary objectives, with differences in tumor cell sources, culture techniques, and formation time. Organoids can be cryopreserved and cultured for an extended period. Genetically and histologically, organoids can mimic the original tissue. Additionally, organoids are susceptible to genetic manipulation and can be produced from tiny amounts of tissue [23,24]. These features make them applicable to various applications in cancer research, including drug development, carcinogenesis research, and personalized treatment. The properties of organoid and spheroid are depicted in Figure 1.

### Fabrication and Characterization Technique for 3D Spheroids

Spontaneous aggregation is the easiest way to form cellular spheroids, in which cells spontaneously cluster to form 3D cell aggregates. Different techniques like matrix embedding, spinner flasks, ultra-low attachment plates, micro-patterned plates, magnetic levitation, magnetic 3D printing, hanging drop, matrix on top, and matrix encapsulation have been developed over the years to generate spheroids [25]. The advantages and disadvantages of each technique are listed in Table 3 [25,26,27,28,29,30,31].

Many methods are currently used to characterize the features of 3D tumor spheroids, including (i) topography, (ii) morphology, (iii) size, (iv) metastatic potential and invasiveness, (v) gene and protein expression, (vi) cell cycle patterns and (vii) cellular organization of cancer cells. These approaches have also been used to describe the influence of 3DS and cell death on anticancer therapies [32].

**Figure 2:**
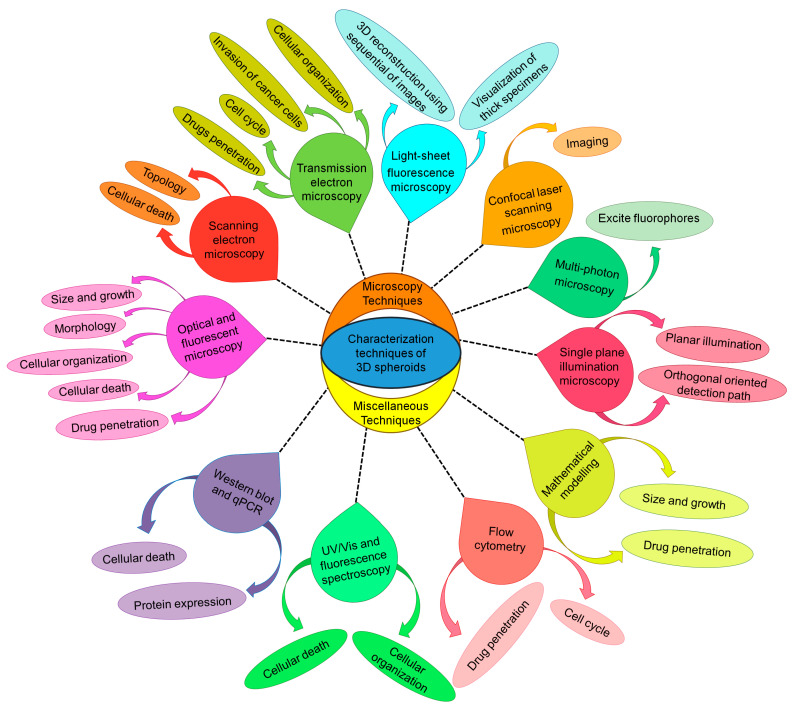
Characterization techniques for 3D tumor spheroids.

Different techniques have been explored for the establishment of histological analysis of 3D tumor spheroids, such as optical and electron microscopy, flow cytometry and Western blotting, ultraviolet–visible spectroscopy (UV/Vis) and fluorescence spectroscopy, UV/Vis and fluorescence spectroscopy, multi-photon microscopy, and confocal laser microscopy (Figure 2).

## 4. Use of 3D Spheroid Models to Investigate Different Cancers

The intricate microenvironment in which malignant cell reside is essential for the progression of tumor growth. The biochemical as well physical properties of TME are necessary for the proliferation, metastasis, and invasion of cancer cells. Consequently, it is essential to ascertain how malignant cells interact and communicate with supporting tumor-associated cells like endothelial cells, immune cells, macrophages, and fibroblasts. Three-dimensional spheroid models are commonly used to explore the complicated mechanisms behind cancer progression because they simulate the stromal milieu and multicellular structure of an in vivo tumor. Compared to 2D systems and animal models, the 3D spheroid model delivers more accurate information regarding tumor features, drug discovery, cell–cell interactions, and the metabolic profile of cancer cells [33].

In the following paragraph, we will describe a few recent applications of 3D spheroids in cancer research, analyzing the most representative publications.

### 4.1. Prostate Cancer

Prostate cancer (PCa) is a leading cause of death among men worldwide. The 3D cell cultures allow the functions of living tissue to be mimicked and provide essential information coded in tissue architecture. The crucial role of epithelial–mesenchymal transition (EMT) has been considered in cancer development [34,35].

Regarding the cell lines used for 3D models of prostate cancer, Xu et al. [36] designed a porous chitosan-alginate (CA) scaffold for tissue engineering and analyzed the impact of scaffold stiffness on 22Rv1, PC-3, and C4-2B cell lines. CA scaffold is a 3D culture technology that facilitates phenotypic expression and PCa development with long-lasting scaffold stiffness, mimicking the metastatic advancement phase. 22Rv1 and C4-2B cells (androgen receptor positive) developed multicellular spheroids, while PC-3 cells (androgen receptor negative) formed only clusters.

To design a chemotherapeutic screening tool, PCa cells were co-cultured with fibroblasts. Along this line, Fontana et al. [37] explored the impact of the 3D structure on the development of some primary EMT markers in cultured human DU145 and PC3 cells in 2D monolayers or 3D spheroids. Authors found that several EMT markers, like E-cadherin, are more expressed in 3D spheroids than in 2D monolayers.

This finding helps to understand the role of EMT in PCa and indicates that a 3D model of cell culture may provide further knowledge in cancer biology.

### 4.2. Liver Cancer

The use of 3D spheroid culture of hepatocellular carcinoma (HCC) cells is promising for understanding tumor–TME interactions and the mechanistic details of chemotherapeutic resistance [38].

Hepatic carcinoma-derived cell lines, like HepG2, C3A, HepaRG, and HuH6, are widely used due to their unlimited growth, availability, and high reproducibility of results. For a better understanding of genotoxicity, Stampar et al. [39] developed a HepG2 3D spheroid model and analyzed the mRNA expression profile of genes coding for cell proliferation, drug-metabolizing enzymes, transporters, and liver-specific factors. The findings showed a time-dependent reduction in cell proliferation, with cell division arrested in both the non-proliferating and proliferating phases of the cell cycle. Furthermore, the spheroids showed improved liver-specific activities as well as substantial physiological significance regarding gene expression of hepatic markers and metabolic enzymes.

The main message is that the initial cell density for spheroid formation is essential in order to produce spheroids with viable dividing cells, a prerequisite for investigating the adverse geno-/toxic effects.

In conclusion, HepG2 3D spheroid models provide a reliable assessment of the genotoxic activity of chemicals and may provide an alternative to animal models.

### 4.3. Breast Cancer

Triple-negative breast cancer is a highly aggressive form of breast cancer with few therapeutic options since it lacks estrogen and progesterone receptors as well as human epidermal growth factor receptor 2 (HER-2). Altered metabolic pathways are one of the hallmarks of breast cancer, while the concentration of nutrients plays a significant role in the metabolic process of cancer cells.

Bizjak et al. [40] used MDA-MB-231 breast cancer cell lines to analyze the effect of glucose, pyruvate, and glutamine on the metformin metabolic reaction in both a 2D monolayer culture model and a 3D spheroid model. The findings showed that the non-essential amino acids inhibited the effect of metformin on MDA-MB-231 cells in both the 2D culture model and the 3D spheroid model. Glutamine and pyruvate weakly diminished the effects of metformin in 2D culture. Under glucose-depleted conditions, metformin suppressed the proliferation of MDA-MB-231 cells, disintegrated tumor spheroids, and reduced cell survival.

The key message is that glucose is probably the major carbon source to sustain the proliferation of metformin-treated cells. As a result, it is reasonable to believe that MDA-MB-231 cells treated with metformin rely on glutamine metabolism only to a limited extent.

According to the above findings, researchers should examine the source of nutrients when analyzing the effectiveness of metformin in 2D culture and biologically more relevant 3D tumor spheroids.

### 4.4. Pancreatic Cancer

Pancreatic ductal adenocarcinomas (PDACs) are considered morphologically and functionally heterogeneous. Genetic, transcriptional, and morphological abnormalities have been reported, while researchers found that epithelial or mesenchymal features were more enhanced in 3D cancer models than in 2D models.

Minami et al. [41] investigated the morphological and functional differences between eight PDAC cell lines in 2D and 3D cultures.

They found, in 2D cultures, that most PDAC cells exhibited comparable pleomorphic morphologies. PDAC cells with high E-cadherin and low vimentin expression levels (epithelial) formed small round spheres surrounded by flat-lining cells in 3D culture, whereas those with high vimentin and low E-cadherin expression levels (mesenchymal) formed large grape-like spheres without lining cells and were highly proliferative.

In conclusion, the 3D-culture method can be used to investigate the diversity of PDAC cell lines and may play a significant role in developing customized early detection methods and anticancer drugs for PDAC.

### 4.5. Thyroid Cancer

Thyroid cancer incidence has increased globally in recent years because of the high population awareness of screening programs, increased laboratory testing and identification in imaging examination, and more accurate diagnostic methods [42].

Oh et al. [43] studied the expression of thyroid differentiation proteins related to iodide-metabolizing pathways in thyroid cancer cells under various culture conditions. One cell line from the thyroid follicular epithelium (Nthy-Ori 3-1) and four (BCPAP, BHP10-3SCp, K1, and TPC-1) from thyroid cancer were grown on agarose-coated plates in 2D adherent cell culture and 3D spheroid culture.

They found that the proliferation in 3D spheroids was significantly reduced, whereas hypoxia-inducible factor-1 (HIF-1) was upregulated. Moreover, 3D spheroids with thyroid cancers exhibited diminished thyroid differentiation markers, whereas thyroid follicular epithelial cells exhibited either a stable or significant decline in protein expression.

Due to cellular proliferation, hypoxia, ECM, morphology, viability, thyroid differentiation, and cytoskeleton changes, researchers confirmed that the 3D spheroid culture environment could mimic in vivo environments.

### 4.6. Lung Cancer

An estimated 1.6 million deaths/year from lung cancer have been recorded globally, with a 10% survival rate in the last five years. Among this, more than 80% of cases are from non-small-cell lung cancer (NSCLC).

Chauhan et al. [44] investigated the in vitro efficacy of inhaled erlotinib nanoemulsion in the NSCLC A549 cell line.

In this study, the IC50 for the erlotinib-loaded nanoemulsion was 2.8 times lower than that of the erlotinib-free solution. In addition, ex vivo experiments utilizing a 3D spheroid model demonstrated that erlotinib-loaded nanoemulsion is more effective against NSCLC.

Therefore, synthesized nanoemulsion has the potential to be a promising therapy against NSCLC that can be nebulized locally into the lungs.

### 4.7. Ovarian Cancer

Ovarian cancer (OC) is a significant issue, with a five-year survival rate of about 40%. This is due to the lack of evident and consistent symptoms at the beginning of the disease, which causes more than 80% of patients to be detected at severe stages. More relevant in vitro models that mimic the complexity of the OC microenvironment and the dynamics of the OC cell population are needed to understand OC pathophysiology better and improve drug screening. Recent advances in 3D cell culture and microfluidics have enabled the development of highly novel models capable of bridging the gap between pathophysiology and mechanical models for clinical research [45].

Fiegl et al. [46] analyzed OPRM1 expression, the main receptor and action site of methadone, in OC cell lines and OC tissues. They also investigated pro-angiogenetic, cytotoxic, and apoptotic effects of D,L-methadone in OC cell lines (A2780 A2780Cis, HTB77, OVCAR3, SKOV6, and HOC7) and four patient-derived tumor-spheroid models.

Only OVCAR3 showed OPRM1 expression out of eight at the mRNA and protein level, whilst, in 69% of the analyzed OC tissues, OPRM1-mRNA was detected at a very low level without protein expression. Irrespective of OPRM1 expression, D, L methadone treatment dramatically reduced cell viability in five OC cell lines (SKOV6, OVCAR3, A2780, A2780 Cis, and M019i). D, L-methadone, alone or in combination with cisplatin, had no effect on apoptosis or VEGF secretion in cell lines. There was a significant increase in cell proliferation in two of the four spheroid models after prolonged exposure to D L-methadone, while inhibitory effects of cisplatin in three spheroid models were observed after the addition of D,L-methadone.

In conclusion, the expression of OPRM1 is not necessary for D,L-methadone function in all OC samples. As a result, D,L-methadone may also have negative consequences by promoting the proliferation of certain OC-cells and countering the therapeutic effects of cisplatin.

## 5. Three-dimensional Spheroid-Based Theragnostic Applications in Cancer Drug Discovery

Three-dimensional spheroid-based theragnostic applications are receiving increasing worldwide attention due to their application in several different therapies. As examples, in the following paragraph, we will describe some recent applications of 3D spheroids for cancer therapy.

### 5.1. Nuclear Medicine Therapy

Nuclear medicine is a multidisciplinary field that studies physiological processes and uses radiopharmaceuticals to diagnose and treat diseases non-invasively. Three-dimensional models are increasingly being used in radiopharmaceutical research. The primary goal is to characterize novel radiotracers in vitro for nuclear medical imaging using single photon emission computed tomography (SPECT) or positron emission tomography (PET) [47,48,49,50,51,52,53]. Furthermore, 3D models can be used to design targeted nuclear medicine therapy (e.g., effects of α or β labelled radiopharmaceuticals). Table 4 lists the recently developed radiopharmaceuticals using 3D models of various tumor types [54,55,56,57,58,59,60,61,62,63].

Fluorodeoxyglucose PET ([^18^F]FDG-PET) is commonly used to monitor the therapy response and provide an early indication of the long-term response. This is predicated on the concept that glucose consumption changes correlate with viability and long-term growth [64,65,66,67,68,69].

As an example, Kelly et al. [70] investigated the impact of the PI3K inhibitors (NVP-BEZ235 and NVP-BKM120) on [^18^F]FDG uptake and its relationship with 3D growth using multicellular tumor spheroids. FaDu (human nasopharyngeal) and EMT6 (mouse mammary carcinoma) cell lines have been used to form spheroids. They found that growth was considerably inhibited (*p* < 0.0001) in a dose-dependent manner in spheroids from both cell lines treated with either inhibitor. In the highly proliferative cell line EMT6, [^18^F]FDG uptake was significantly reduced in EMT6 at all concentrations of inhibitor, while in the FaDu, [^18^F]FDG uptake was affected dose-dependently but to a lesser extent.

This study indicates that [^18^F]FDG can be an appropriate marker of response to PI3K inhibition in the investigated cell lines.

Another example comes from neuroendocrine tumors (NETs). These are a heterogeneous family of neoplasms that develop from enterochromaffin cells of the diffuse neuroendocrine system. One of the most promising targeted therapeutics for neuroendocrine tumors (NETs) is peptide receptor radionuclide therapy (PRRT) with ^177^Lu-octreotate (^177^LuTate), but it rarely achieves complete remission. So, Adant et al. [71] investigated various strategies to enhance the efficacy of ^177^Lu-Tate PRRT in NET patients.

They used 2D and 3D cell culture models of human-derived GEP-NET and BP-NET cell lines to show that PARPi potentiates the therapeutic efficacy of ^177^LuTate PRRT in NET. In more detail, PARPi improves PRRT in human NET cell lines by enhancing the downstream effects of ^177^LuTate-induced DNA damage, including cell cycle arrest and apoptosis. Since several PARP inhibitors, including olaparib, are already being used in the clinic to treat other malignancies, combining PARPi with ^177^Lu-Tate could offer a new possibility for improving the efficacy of PRRT in patients with NET.

Finally, radiolabeled nanoparticles (NPs) are a potential nuclear medicine technology for diagnostic and therapeutic purposes. Magnetic nanoparticles (NPs) can be used in nuclear medicine to diagnose and treat [72,73,74]. Unak et al. [75] used superparamagnetic iron oxide (SPION) NPs labeled with two scandium radionuclides, ^44^Sc and ^47^Sc, for detecting and treating prostate cancer. A 3D spheroid model has been developed using the LNCaP (PSMA+) and PC-3 (PSMA−) prostate cancer cell lines. The radio-bioconjugate showed much greater affinity and cytotoxicity for LNCaP (PSMA+) human prostate cancer cells than for PC-3 (PSMA-) cells. Radiotoxicity investigations on LNCaP 3D spheroids validated the high cytotoxicity of radio-bioconjugate. The authors concluded that this investigation showed that a stable magnetic PSMA radio-bioconjugate tagged with ^44^Sc and ^47^Sc could be used to treat aggressive prostate cancer.

### 5.2. Stem Cell Therapy

Cancer stem cells (CSCs) are distinguished by an improved self-renewal potential, the ability to seed novel tumors, and chemo-resistance [76,77]. Different strategies convey the progress of CSC-targeting drugs, including (i) patient sample inadequacy, (ii) physiological relevance of the culture platform, (iii) drug sensitivity differs amongst patients, and (iv) difficulty in expanding and maintaining CSCs in vitro [78]. CSC research in 3D models could help us better understand carcinogenesis, tumor growth, metastasis, and recurrence in vivo and contribute to possible drug discovery for the treatment of tumors.

As an example, to mimic the in vivo situation as closely as possible, Wessely et al. investigated the effect of bone marrow-derived mesenchymal stem cells (BMSCs) from four different donors combined with four head and neck squamous cell carcinoma (HNSCC) cell lines in a 3D spheroid model [79,80] and also analyzed the gene and protein expression of several markers that are typically involved in chondro-, osteo-, and adipogenic differentiation of mesenchymal stem cells (MSCs). Results demonstrated that the two osteogenic markers RUNX2 and ALPL were up-regulated in heterogeneous BMSC/HNSCC spheroids, implying that direct interaction between HNSCC and BMSCs leads to BMSC priming toward the osteogenic lineage, leading to increased invasive behavior of the tumor cells [81].

This research provides new perspectives on the intricate relationship between HNSCC and its tumor stroma, which could help in the development of novel therapies to prevent HNSCC disease progression.

### 5.3. Photodynamic Therapy

Photodynamic therapy (PDT) generally consists of three distinctly harmless elements, such as photosensitizers (PS), followed by light and oxygen, which promote cell damage. PDT has already been widely studied for clinical applications like cancer treatment and other diseases like posterior capsule opacification and age-related macular degeneration [82,83].

Dobos et al. [84] developed a two-photon (2P) excited photosensitizing agent (TPE-PS) pre-screening platform employing a 3D osteosarcoma (MG63 cells) model with adipose tissue-derived stem cells (ASC/TERT). Three different two-photon (2P) active substances like porphyrin derivative (TPP), fluorescent dye Eosin Y, and a 2P sensitizer P2CK were used to test this developed system. Their findings showed a 65% and 75% cell viability reduction after 2P irradiation in the presence of P2CK and TPP, respectively. This pre-screening method allows for high-throughput profiling of TPE-PS and a comprehensive investigation of PS efficacy in vitro.

Another example was reported by Kumari et al. [85] They developed a self-assembled amphiphilic polymer, chlorine e6-conjugated methoxy-poly (ethylene glycol) poly (D, L-lactide) (mPEG-PLA-Ce6), to form stable NPs. Cellular internalization and phototoxicity of human lung adenocarcinoma cells (A549) were studied in monolayer and 3D spheroids. A time-dependent cellular internalization uptake of mPEG-PLA-Ce6 was observed, while the phototoxicity of mPEG-PLA-Ce6 to A549 cells increased significantly when compared to free drug, which could be attributed to precise cellular uptake and rapid release of Ce6 from nanoparticles. This proposed delivery system was able to transport Ce6 to cancer cells more efficiently while also imparting stability and increasing phototoxicity. As a result, the Ce6 micellar system developed has the potential for efficient cytosolic delivery of PSs for photodynamic therapy of solid tumors.

### 5.4. Immune Therapy

Finally, immune checkpoint inhibitor therapy has improved clinical practice for patients with various cancers, as these inhibitors have shown substantial overall survival progress and are successful in many cases. Additionally, inherent or acquired resistance also exists, and predictive biomarkers of sensitivity can aid in selecting patients and determining the appropriate treatment options. The 3D cell culture models mimic the landscape tumor microenvironment and screen immunomodulatory drugs [86].

Herter et al. [87] explored the adaptability of a 3D heterotypic spheroid model composed of tumor cells, fibroblasts, and immune cells by investigating drug targeting and immune cell infiltration, activation, and cytotoxicity in response to novel cancer immunotherapy agents (IgG-IL2v and T-cell-bispecific antibodies (TCBs)) used alone and in combination. T, NK, and NKT cell activation was indicated by increased expression of CD69 marker and increased cytokine secretion after IgG-IL2v therapy. The combination of TCBs with IgG-IL2v molecules surpassed monotherapy by enhancing immune cell infiltration and activation as well as faster, more efficient removal of targeted cells and cytokine release. This study demonstrates that the 3D heterotypic spheroid model is a novel and versatile tool for in vitro evaluation of cancer immunotherapy agents, allowing for the analysis of immune cell infiltration and drug targeting.

## 6. Nanocarriers in 3D Spheroids Model

There has been a significant increase in innovation in the field of nanomedicine, primarily focusing on the development and analysis of specifically engineered carrier systems to transport payloads of therapeutic and diagnostic agents to achieve the target and sustain delivery [88,89,90,91]. Nanoparticles (50–200 nm) prevent off-target effects by providing a longer circular duration, discharging active ingredients at a target site in a controlled manner, and successfully translocating across cell membranes. Furthermore, the modern, diverse chemical composition materials utilized in the production of nanoparticles allow for the encapsulation of diagnostic and therapeutic agents with different physicochemical properties [92,93]. For such advanced nanocarriers, this less-than-stellar commercial performance may be due to the difficulties encountered during their classification and, ultimately, consistent development in their bulk size. Conventional 2D cell culture models evaluate the efficacy of NP in early growth, but transitioning from such over-simplified models, typically with over-promising findings, to more intricate in vivo conditions takes time. Information on the cellular interaction of NPs can be obtained using in vitro cell culture models. In contrast, animal models can be used to extract evidence on the effectiveness and toxicity of NPs. However, there needs to be more knowledge about the association of NPs with tissue components and structures like cells, ECMs, and other physiological variables that 3D tissue models can measure. Emerging drug-intensive nanomedicines are a positive path to solving these issues [94,95,96]. Nanocarriers solubilize, avoid degradation, and enhance the biopharmaceutical properties of the drug through encapsulation, facilitate long drug diffusion, and increase stability. In comparison, drug-loaded nanocarriers show improved permeability and retention effects, which enhance the drug concentration at the tumor site compared to the normal tissues while improving the effectiveness and toxicity of the loaded drug [97,98].

Multicellular tumor spheroids (MCTS) are now recognized to be more accurate than 2D cell-culture-based assessments for high-throughput drug screening. This approach enables the identification of negative and positive new drug possibilities, notably nanotherapy, to eliminate the need for animal testing [99,100,101]. Patra et al. recently established the efficacy of flow cytometry pair microfluidics for the rapid development and viability evaluation of a large number of well-defined sizes MCTS exposed to and coupled with various drugs [102]. When assessing the capacity of NPs to be useful in vivo, the relationship between the ability of NPs to accumulate/penetrate the spheroid and drug cytotoxicity is considered [103,104,105].

Table 5 summarizes recent research using the MCTS model to test compound accumulation, penetration, and cytotoxicity to show the anticancer ability of novel nanotherapeutics in this background. In general terms, with NPs displaying high penetration and aggregation, the highest cytotoxic effect was achieved in MCTS. The DOX, one of the most widely used drugs in oncology, has played a vital role in many nano-formulations, either approved for patient care or undergoing clinical investigations [106]. The significant adverse effects caused by DOX inpatients are, in fact, a considerable argument for increasing the targeted distribution of DOX to tumors (Table 5) [104,107,108,109,110,111].

### 6.1. Dendrimers

The most important feature of dendrimers is transferring bioactive materials such as drugs, genes, vaccines, and metals to the specified location [112,113,114,115]. Due to the hollow space found within dendrimers, narcotics and other bioactive are loaded into it using physical or chemical approaches and act as a medium for the distribution of drugs. Different dendrimers such as poly(amidoamine) (PAMAM, i.e., Starburst^TM^), poly-L-lysine (PLL), poly (propylene imine) (PPI) and Triazine, have broadly been discovered as gene and drug delivery carriers for tumor imaging worldwide for enhanced diagnosis and cancer treatment [112,113,116,117].

Rompicharla et al. [118] developed generation 4 (G4) PAMAM dendrimers to deliver a poorly soluble anticancer agent like PTX to cancer cells, specifically through its dendrimer surface conjugation. The conjugates were tested in vitro in cell monolayers and the 3DS of biotin receptor over-expressed cell line A549 (human non-small cell lung cancer).

The G4-PTX-PEG-biotin conjugate had considerably higher penetration in monolayers as well as in spheroids. The G4 PTX PEG-biotin conjugate demonstrated higher cytotoxicity compared to free. The G4-PTX-PEG-biotin showed significant inhibition of tumor spheroid development. The recently synthesized PTX-conjugated dendrimer device anchored biotin is encouraging and can be investigated to effectively provide PTX to over-expressed cancers with biotin receptors.

### 6.2. Quantum Dots

Quantum dots (QDs) introduce new perspectives into the theragnostics of cancer. In conjunction with the easy probability of altering the surface with guiding molecules, exceptional visibility allows QDs to be used as enticing agents in fluorescence-guided surgery and photodynamic therapies [119,120]. Some targeted QDs are currently being established for theragnostic purposes. Nevertheless, their targeting ability was evaluated mainly in tumor cell models of a 2D monolayer.

Mangeolle et al. [121] demonstrated the ability of folic acid (FA)-conjugated QDs to target tumors in a spheroid model (KB cell spheroid model), thus validating the essential function of the FRα receptor as a target. The findings confirmed the specificity of QD-FA for the folic acid receptor-positive KB cells. In a 3D tumor spheroid model, the QD-FA uptake was enhanced when compared to nontargeted QD.

### 6.3. Carbon Nanotubes

Carbon nanotubes (CNTs) have been explored as promising nanocarriers for the delivery of bioactives [122,123,124,125]. It has been shown that multi-walled carbon nanotubes (MWCNTs) induce the development of pro-fibrotic and inflammatory mediators and histopathological modifications in infected animal lungs. It has been shown that 3D in vitro models recapitulate human physiology more reliably than conventional 2D in vitro or in vivo animal models, offering a new, more reliable approach to assessing chronic and acute toxicity in a structured nanomaterial toxicity research system.

Since inhalation is a significant route of exposure to nanomaterials; Kabadi et al. [126] developed scaffold-free 3D lung microtissues by culturing human lung fibroblasts (IMR-90) and epithelial cells (BEAS-2B) with macrophages (primed THP-1 monocytes). These microtissues were exposed to M120 carbon black NPs, MWCNTs, or crocidolite asbestos fibers for 4 or 7 days. The study outcomes demonstrated that the application of 3D microtissues can predict chronic pulmonary endpoints by exposure to asbestos fibers or MWCNTs. In conclusion, 3D lung microtissues provide a significant method of investigation for assessing the development of nanomaterial-induced cell-matrix and identifying toxicity pathways, paving the way for a more precise and physiologically appropriate approach to in vitro NP toxicity monitoring.

### 6.4. Liposomes

Liposomes have revolutionized cancer therapy due to their broad clinical applications. Liposomes resolve the restrictions of traditional chemotherapy by enhancing the bioavailability and stability and minimizing side effects through site-specific targeted delivery of drugs [127].

Rodallec et al. [128] developed 3D spheroid models to test the efficacy of trastuzumab docetaxel (DTX) immune liposomes in breast cancer. Two breast cancer cell lines, MDA-MB-453 mammary breast cells and MDA-MB-231 triple-negative cells, were evaluated. To assess the viability, fluorescence detection of 3DS was developed and tested. Tumor growth was reduced by 66% (MDA-MB-453) and 29% (MDA-MB-231) relative to T-DM1 and 89% (MDA-MB-453) and 25% (MDA-MB-231) compared to free DTX + free trastuzumab. These findings conclude that immunoliposomes can achieve higher efficacy than reference treatments (i.e., free docetaxel + trastuzumab or T-DM1), most likely due to improved drug delivery based on passive and active targeting.

### 6.5. Polymeric Micelles

Polymeric micelle, the self-assembled amphiphilic block copolymer produced in an aqueous solution, has an inner hydrophobic center and an outer hydrophilic shield, also identified as a corona [129]. These act as a primary vector for the transmission of anticancer drugs and, due to their core–shell configuration, may imitate the biological transport system [130,131].

Kumari et al. [132] developed a nano-formulation that encapsulates Ce6 in the copolymeric micelles of methoxy-poly (ethylene glycol) poly (D, L-lactide) (mPEG-PLA). The therapeutic efficacy of Ce6-mPEG-PLA micelles after exposure was tested in vitro in 2D and 3D cell culture systems utilizing human uterine cervical cancer (HeLa) and human alveolar adenocarcinoma (A549) cells in monolayers and spheroids, respectively. Compared to free Ce6, the Ce6-mPEG-PLA micelle-mediated PDT showed improved cellular uptake and cytotoxicity in both cell types. The Ce6-loaded micelles penetrated deeply into the spheroids, causing phototoxicity and cell death in the A549 spheroidal model. These findings suggested that the newly synthesized nano-formulation of Ce6 could be used in PDT as a successful therapeutic strategy for solid tumors.

### 6.6. Silver Nanoparticles

Compared with other metallic NPs such as magnesium, iron, zinc, copper, and titanium, silver nanoparticles (AgNPs) have been proven to be the most potent antimicrobial agents [133]. However, when used as a product disinfectant, AgNPs present many risks as they induce toxicity in mammalian cells, and due to exposure to platinum, argyrosis and argyria are induced.

Arora et al. [134] developed glioblastoma U-87 MG and breast cancer MCF7 spheroids to assess the therapeutic prospect of recombinant phosphatase and tensin homolog (PTEN) protein. The efficacy of PTEN-nanocomposites on MCF7 and U-87 MG spheroids indicated successful control of the cellular environment, resulting in cell cycle arrest, gene expression regulation, and reduced spheroid proliferation. In 3D spheroid cells, greater dosages of recombinant PTEN protein are required to achieve a similar effect as in monolayer cultures, indicating the complexity of the 3D spheroid cultures.

### 6.7. Nanogels

Nanogels are revolutionary hydrogel structures containing cross-linked polymers of natural or synthetic origin with a strong capacity to hold water. The hydrophilic structure of nanogel has good water absorption and exhibits regulated and sustained release. Nanogels demonstrate promising characteristics such as high biodegradability, biocompatibility, capacity to load drugs, and good penetration power [135,136].

Cheng et al. [137] developed D-α-Tocopheryl polyethylene glycol succinate (TPGS)-grafted and acid-responsive soybean protein (SP)-based nanogels for effective intracellular drug release and accumulation in A549 and A549/DDP cells. A series of 2D and 3D cell evaluations confirmed that TPGS-modified nanogels can enhance cellular uptake and drug accumulation, resulting in a more significant antitumor effect in drug-resistant cells. These findings conclude that smart SP-based nanogels have a high potential for more efficient and long-lasting drug treatment in cancer cells, particularly in overcoming multidrug resistance in solid tumors.

### 6.8. Nanodiamonds

Nanodiamonds find new and far-reaching uses in contemporary biomedical sciences and biotechnology. Because of their superior biocompatibility, nanodiamonds act as flexible frameworks that can be incorporated into microfilm applications centered on polymers. Nanodiamonds complexed with a chemotherapeutic allow the drug to be released sustainably and slowly for at least one month, with a considerable amount of the drug in reserve [138].

Madamesetty et al. [139] developed DOX-loaded PEG-functional nanodiamonds (ND-PEG-DOX), which substantially improved the free drug in an orthotopic pancreatic xenograft model. ND-PEG-DOX showed significantly more uptake when compared to free DOX in both BxPC3 and PANC-1 cell lines. A superior cytotoxic effect was also recorded when we compared ND-PEG-DOX to free DOX in a 3D tumor spheroid model of PDAC. These findings conclude that ND-mediated drug delivery can enhance therapeutic outcomes in PDAC.

### 6.9. Polymeric Nanocarrier

Because of some intrinsic properties such as non-immunogenicity, nontoxicity, biocompatibility, and biodegradability, polymeric NPs are used in nanostructures as an alternative approach [140]. The biodegradable polymer poly(lactic-co-glycolic) acid has been shown to have significant potential as a drug delivery vehicle [141,142,143,144,145].

Le et al. [146] designed docetaxel-loaded pegylated poly(D, L-lactide-co-glycolide) NPs conjugated with anti-HER2 single-chain antibodies (scFv–Doc–PLGA–PEG) and evaluated the cellular uptake and cytotoxic effect of scFv–Doc–PLGA–PEG on a 3D tumor spheroid model of BT474 (HER2-overexpressing) and HCT116 (HER2-underexpressing) cancer cells. Compared to HER2-underexpressing cancer cell spheroids, the nanoparticle formulation coupled with scFv had a considerable internalization effect on HER2-overexpressing cancer cell spheroids. Thus, the cytotoxic effects of targeted nanoparticles diminished the size and increased the necrotic score of HER2-positive tumor spheroids. This drug delivery system has been presented as a possible method for improving the efficacy of nanoparticles in active targeting for HER2-overexpressing cancer therapy.

### 6.10. Nanozymes

Some constraints substantially limit the broad usage of enzymes. Enzymes are proteins or RNA rapidly degraded by ribonuclease and protease, making them difficult to transport and store. Environment-sensitive enzymes can catalyze specific reactions due to their different three-dimensional architectures. Therefore, enzymes are quickly denatured and inactive when exposed to severe circumstances such as strong acids/bases and high temperatures. Enzymes are often extracted from living cells, resulting in lengthy and expensive purification and separation operations. The fabrication of artificial enzymes is currently being investigated as a potential solution to these problems. Nanozymes, with their capacity to mimic enzymes and nanoscale size, have risen to prominence in artificial enzymes. Nanozymes are nanomaterials that use enzyme kinetics to catalyze chemical reactions under physiological conditions requiring enzyme substrates [147]. Like regular enzymes, nanozymes possess great catalytic activity and can accelerate biological activities. Nanozymes often have simple preparation/purification processes, minimal production costs, and good stability due to their nanomaterial composition.

Valho et al. [148] developed a peroxidase-like (POD) nanozyme for biocatalytically destroying glioblastoma (GBM) cancer cells based on a 3D spheroid model.

The results indicated that these nanozymes inhibited tumor growth and dramatically reduced malignant tumor volume (~40%following nanotherapeutic therapy. The kinetics of anticancer activity of these novel nanotherapeutic agents decreased with the incubation period of the GBM 3D models, demonstrating a trend frequently observed in TMEs.

## 7. Conclusions and Future Perspectives

Animal models are primarily used in the laboratory to observe the therapeutic effects of drugs for anticancer treatment. Currently, the application of 3D models is gaining more attention in clinical research. Compared with 3D cell cultures, spheroids are more effective because of their reproducibility, ease of handling, and economic properties, which can help the large-scale production of cellular aggregates for high-throughput screening of pharmaceuticals. The 3DS system can precisely describe the in vivo cancer microenvironment, enabling the exploration of the fundamental biological mechanism associated with metastasis and primary tumors, and helps to analyze the therapeutic effects of nanocarriers, cell targeting efficacy, and related nanotoxicity. Various research data demonstrate that spheroids may represent the most effective model to characterize solid tumors. Improving the methodology of spheroid development and further exploring the microenvironmental biology of tumors is essential in promoting the correlation between spheroid models and the human body.

## Figures and Tables

**Table 1:** A comparison between three different cultural models.

Culture Models	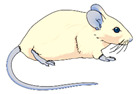 PDX Animal Model	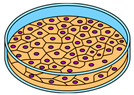 2D Model	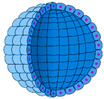 3D Model
Ethical and regulation issues	Yes	No	No
Model complexity	Very complex	Complex	Intermediate
Physiological relevance	High	Intermediate	More than 2D
Reproducibility	Unsuited	High	Less than 2D
Data provider	Difficulty in exploit	Easy exploit	Easy exploit
Drug screening	Less effective	Highly effective	Effective
Controlled microenvironment	No	Yes	Yes
Mimicking the original tumors	Intermediate	Less	Intermediate
Preservation of tumor morphology	Intermediate	Less	Less
Success rate of model generation	Less	Less	Intermediate
Maintenance	High	Less	Intermediate
Cost	High	Less	Intermediate

**Table 2:** Benefits and associated risks between scaffold-based and scaffold-free systems.

3D Tumor Models	Formulation Technique	Benefits	Associated Risks	References
Scaffold-based systems 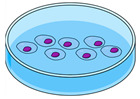	Seeding of cells on an acellular 3D artificial matrix Cell dispersion in hydrogels	Personalized Co-cultures possible The large variety of materials also decellularized matrix A large number of cell-cell interactions take place	External biomaterials are required ECM is artificial Cell removal may be difficult No cell/extracellular matrix (ECM) interactions Not always optically transparent Limited high-throughput screening (HTS) options Expensive	[7,8]
Scaffold-free systems 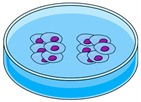	Microfluidic Hanging drop Liquid overlay Spinner flask Magnetic bioprinting	No external biomaterials are required ECM is produced by the cells HTS approach possible Co-cultures possible Optically transparent The majority of techniques are inexpensive A large number of cell-cell interactions take place	For the formation of uniform spheroids, optimizations may be required Poor control over spheroids No porosity	[9,10]

**Table 3:** Different 3D spheroid fabrication techniques and their advantages/disadvantages.

Techniques	Advantages	Disadvantages	References
Hanging drop technique (HDT) 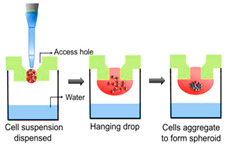	Simple and economic Spheroids can be formed in distinct compartments Special equipment not required The shape and size of the spheroids are reproducible	Not stable Laborious Spheroids can hold large volumes of media due to long-term culturing	[26,27]
Spinner flask 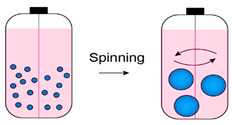	Mass production High throughput Simple	Expensive Nonhomogeneous size and cell composition	[28,29]
Magnetic levitation and magnetic bio-printing 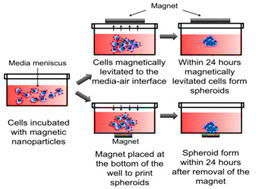	Imaging and other biochemical assays can be multiplexed. On the same plate, endpoint analysis can be performed.	Limited numbers of spheroids Beads at high concentrations might be toxic to cells Beads are expensive	[28,29]
Liquid overlay technique (LOT) 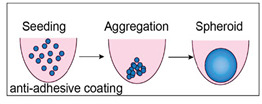	No need for specific equipment Simple and economic Specific compartments should be used for each spheroid	To obtain spheroids of uniform size and shape, optimization is required. The method of plate preparation can be laborious.	[30]
Microfluidics 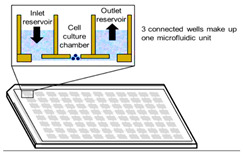	Rapid formation of spheroids Continuous perfusion The shape and size of the spheroids are reproducible The capacity to monitor the exchange of nutrients and gases	Spheroid extraction can be difficult Manufacturing of the instrument includes specialized materials and equipment.	[31]

**Table 4:** Examples of radiopharmaceuticals utilized in 3D models of various tumor types.

Radionuclide	Conjugation	Targeted Tumor	Result	Ref.
^224^Ra and ^212^Pb	^224^Ra/^212^Pb-TCMC-TP-3 and ^212^Pb-TCMC-TP-3	Osteosarcoma	An 11.4-fold reduction in spheroid viability has been shown in treatment with 1 kBq/mL of ^224^Ra/^212^Pb-TCMC-TP-3 for 24 h compared with unconjugated ^224^Ra/^212^Pb.	[54]
^223^Ra	^223^Ra-hydroxyapatite (HAp) 3DS model	Prostate cancer cells	It generated high levels of apoptosis by inhibiting cell growth irrespective of cell type.	[55]
^90^Y	Cetuximab (C225)	Head and neck squamous cell cancer (HNSCC)	Unconjugated C225 treatment did not affect spheroid development or cell viability.	[56]
^213^Bi	HER-2/neu antigen	Breast cancer	Effective in treating early-stage HER-2/neu--expressing micrometastases.	[57]
^177^Lu	DOTATATE peptide	Neuroendocrine tumors	^177^LuDOTATATE inhibited the growth of BON and NCIH727 spheroids but did not affect NCIH460 spheroids.	[58]
^212^Pb	Monoclonal antibody (mAb) 376.96	Pancreatic ductal adenocarcinoma	PDAC3 cell clonogenic survival was decreased by ^212^Pb-376.96.	[59]
^131^I	ICF01012 MEK inhibitors (MEKi)	Melanoma cells	MEKi combined therapy may be beneficial in treating advanced pigmented BRAF-mutant melanoma.	[60]
^225^Ac	Polymersomes	Glioblastoma	Effectively inhibit tumor spheroid growth	[61]
^131^I	Meta-iodobenzylguanidine (MIBG)	Neuroblastoma	In vivo, ^13I^I-MIBG may spare smaller micrometastases.	[62]
^125^I	Deoxyuridine (IUdR)	Glioblastoma	Nuclear incorporation of [^125^I]IUdR decreased significantly as spheroid size increased.	[63]

**Table 5:** Multicellular tumor spheroids model for testing of drug/bioactive.

Drug/Bioactive	Nanocarriers	Target	Ligand	MCTS	In Vivo Study	Drug Resistance	Ref.
Oregon Green PTX	Liposomes/micelles	Integrin	iRGD peptide	Lung cancer	Negative	Negative	[101]
PTX + CUR + Rhodamine	PEG-phosphatidyl	Tf receptors	Tf	Ovarian cancer	Positive	Positive	[103]
DOX + CUR	Micelles	GLUT1	GLUT1-scFv	Brain cancer U87MG	Negative	Positive	[104]
DOX	Chitosan NPs	Sialic acid groups	CPBA	Brain cancer SH-SY5Y	Positive	Negative	[105]
DOX	Liposomes	Tf receptor	TAT	Brain cancer C6	Positive	Negative	[106]
DOX	PLGA NPs	Tf receptors	Tf	Lung cancer A549	Positive	Negative	[107]

Abbreviations: MCTS—multicellular tumor spheroids; DOX—Doxorubicin; CPBA—4-carboxyphenyl boronic acid; Tf—Transferrin; PLGA—Poly(d,l-lactic-co-glycolic acid); NPs—nanoparticles; CUR—Curcumin; PTX—Paclitaxel; GLUT1—glucose transporter-1 antibody; PEG—polyethylene glycol; TAT—Adenosine-5’-Rp-Alpha-Thio-Triphosphate; scFv—single-chain fragment variable.

## Data Availability

All papers and data are available upon request to P.N.

## References

[1] Jubelin C., Muñoz-Garcia J., Griscom L., Cochonneau D., Ollivier E., Heymann M.-F., Vallette F.M., Oliver L., Heymann D. (2022). Three-Dimensional in Vitro Culture Models in Oncology Research. Cell Biosci..

[2] Jarockyte G., Dapkute D., Karabanovas V., Daugmaudis J.V., Ivanauskas F., Rotomskis R. (2018). 3D Cellular Spheroids as Tools for Understanding Carboxylated Quantum Dot Behavior in Tumors. Biochim. Et Biophys. Acta (BBA) Gen. Subj..

[3] Jamieson L.E., Harrison D.J., Campbell C.J. (2015). Chemical Analysis of Multicellular Tumour Spheroids. Analyst.

[4] Kim J. (2005). Bin Three-Dimensional Tissue Culture Models in Cancer Biology. Semin. Cancer Biol..

[5] Olson B., Li Y., Lin Y., Liu E.T., Patnaik A. (2018). Mouse Models for Cancer Immunotherapy Research. Cancer Discov..

[6] Hinshaw D.C., Shevde L.A. (2019). The tumor microenvironment innately modulates cancer progression. Cancer Res..

[7] Sood D., Tang-Schomer M., Pouli D., Mizzoni C., Raia N., Tai A., Arkun K., Wu J., Black L.D., Scheffler B. (2019). 3D Extracellular Matrix Microenvironment in Bioengineered Tissue Models of Primary Pediatric and Adult Brain Tumors. Nat. Commun..

[8] Yi H.-G., Jeong Y.H., Kim Y., Choi Y.-J., Moon H.E., Park S.H., Kang K.S., Bae M., Jang J., Youn H. (2019). A Bioprinted Human-Glioblastoma-on-a-Chip for the Identification of Patient-Specific Responses to Chemoradiotherapy. Nat. Biomed. Eng..

[9] Boretto M., Maenhoudt N., Luo X., Hennes A., Boeckx B., Bui B., Heremans R., Perneel L., Kobayashi H., Van Zundert I. (2019). Patient-Derived Organoids from Endometrial Disease Capture Clinical Heterogeneity and Are Amenable to Drug Screening. Nat. Cell Biol..

[10] Madoux F., Tanner A., Vessels M., Willetts L., Hou S., Scampavia L., Spicer T.P. (2017). A 1536-Well 3D Viability Assay to Assess the Cytotoxic Effect of Drugs on Spheroids. SLAS Discov..

[11] Shimojo A.A.M., Rodrigues I.C.P., Perez A.G.M., Souto E.M.B., Gabriel L.P., Webster T., Li B., Blanco I. (2020). Scaffolds for Tissue Engineering: A State-of-the-Art Review Concerning Types, Properties, Materials, Processing, and Characterization. Racing for the Surface: Antimicrobial and Interface Tissue Engineering.

[12] Paradiso F., Serpelloni S., Francis L.W., Taraballi F. (2021). Mechanical studies of the third dimension in cancer: From 2D to 3D model. Int. J. Mol. Sci..

[13] Knight E., Przyborski S. (2015). Advances in 3D cell culture technologies enabling tissue-like structures to be created in vitro. J. Anat..

[14] Das V., Bruzzese F., Konečný P., Iannelli F., Budillon A., Hajdúch M. (2015). Pathophysiologically Relevant In Vitro Tumor Models for Drug Screening. Drug Discov. Today.

[15] Fitzgerald K.A., Malhotra M., Curtin C.M., O’Brien F.J., O’Driscoll C.M. (2015). Life in 3D Is Never Flat: 3D Models to Optimise Drug Delivery. J. Control. Release.

[16] da Rocha E.L., Porto L.M., Rambo C.R. (2014). Nanotechnology Meets 3D in Vitro Models: Tissue Engineered Tumors and Cancer Therapies. Mater. Sci. Eng. C.

[17] Simian M., Bissell M.J. (2017). Organoids: A historical perspective of thinking in three dimensions. J. Cell Biol..

[18] Hirschhaeuser F., Menne H., Dittfeld C., West J., Mueller-Klieser W., Kunz-Schughart L.A. (2010). Multicellular Tumor Spheroids: An Underestimated Tool Is Catching up Again. J. Biotechnol..

[19] Holtfreter J. (1944). A Study of the Mechanics of Gastrulation. J. Exp. Zool..

[20] Sutherland R.M., Inch W.R., McCredie J.A., Kruuv J. (1970). A Multi-Component Radiation Survival Curve Using an In Vitro Tumour Model. Int. J. Radiat. Biol. Relat. Stud. Phys. Chem. Med..

[21] Lin R.-Z., Chou L.-F., Chien C.-C.M., Chang H.-Y. (2006). Dynamic Analysis of Hepatoma Spheroid Formation: Roles of E-Cadherin and Β1-Integrin. Cell Tissue Res..

[22] Lancaster M.A., Knoblich J.A. (2014). Organogenesis in a dish: Modeling development and disease using organoid technologies. Science.

[23] Schutgens F., Clevers H. (2020). Human Organoids: Tools for Understanding Biology and Treating Diseases. Annu. Rev. Pathol. Mech. Dis..

[24] Hendriks D., Artegiani B., Hu H., Lopes S.C.D.S., Clevers H. (2021). Establishment of human fetal hepatocyte organoids and CRISPR–Cas9-based gene knockin and knockout in organoid cultures from human liver. Nat. Protoc..

[25] Nath S., Devi G.R. (2016). Three-Dimensional Culture Systems in Cancer Research: Focus on Tumor Spheroid Model. Pharmacol. Ther..

[26] Nunes A.S., Barros A.S., Costa E.C., Moreira A.F., Correia I.J. (2019). 3D Tumor Spheroids as In Vitro Models to Mimic In Vivo Human Solid Tumors Resistance to Therapeutic Drugs. Biotechnol. Bioeng..

[27] Benien P., Swami A. (2014). 3D Tumor Models: History, Advances and Future Perspectives. Future Oncol..

[28] Cui X., Hartanto Y., Zhang H. (2017). Advances in Multicellular Spheroids Formation. J. R. Soc. Interface.

[29] Huang B.-W., Gao J.-Q. (2018). Application of 3D Cultured Multicellular Spheroid Tumor Models in Tumor-Targeted Drug Delivery System Research. J. Control. Release.

[30] Costa E.C., de Melo-Diogo D., Moreira A.F., Carvalho M.P., Correia I.J. (2018). Spheroids Formation on Non-Adhesive Surfaces by Liquid Overlay Technique: Considerations and Practical Approaches. Biotechnol. J..

[31] Moshksayan K., Kashaninejad N., Warkiani M.E., Lock J.G., Moghadas H., Firoozabadi B., Saidi M.S., Nguyen N.-T. (2018). Spheroids-on-a-Chip: Recent Advances and Design Considerations in Microfluidic Platforms for Spheroid Formation and Culture. Sens. Actuators B Chem..

[32] Costa E.C., Moreira A.F., de Melo-Diogo D., Gaspar V.M., Carvalho M.P., Correia I.J. (2016). 3D Tumor Spheroids: An Overview on the Tools and Techniques Used for Their Analysis. Biotechnol. Adv..

[33] Zhu Y., Kang E., Wilson M., Basso T., Chen E., Yu Y., Li Y.R. (2022). 3D tumor spheroid and organoid to model tumor microenvironment for cancer immunotherapy. Organoids.

[34] De Grandis R.A., Dos Santos P.W.D.S., de Oliveira K.M., Machado A.R.T., Aissa A.F., Batista A.A., Antunes L.M.G., Pavan F.R. (2019). Novel Lawsone-Containing Ruthenium(II) Complexes: Synthesis, Characterization and Anticancer Activity on 2D and 3D Spheroid Models of Prostate Cancer Cells. Bioorg. Chem..

[35] Khan R., Arshad F., Hassan I.U., Naikoo G.A., Pedram M.Z., Zedegan M.S., Pourfarzad H., Aljabali A.A.A., Serrano-Aroca Á., Haggag Y. (2022). Advances in Nanomaterial-Based Immunosensors for Prostate Cancer Screening. Biomed. Pharmacother..

[36] Xu K., Ganapathy K., Andl T., Wang Z., Copland J.A., Chakrabarti R., Florczyk S.J. (2019). 3D Porous Chitosan-Alginate Scaffold Stiffness Promotes Differential Responses in Prostate Cancer Cell Lines. Biomaterials.

[37] Fontana F., Raimondi M., Marzagalli M., Sommariva M., Limonta P., Gagliano N. (2019). Epithelial-To-Mesenchymal Transition Markers and CD44 Isoforms Are Differently Expressed in 2D and 3D Cell Cultures of Prostate Cancer Cells. Cells.

[38] Khafaga A.F., Mousa S.A., Aleya L., Abdel-Daim M.M. (2022). Three-dimensional (3D) cell culture: A valuable step in advancing treatments for human hepatocellular carcinoma. Cancer Cell Int..

[39] Štampar M., Breznik B., Filipič M., Žegura B. (2020). Characterization of in vitro 3D cell model developed from human hepatocellular carcinoma (HepG2) Cell Line. Cells.

[40] Bizjak M., Malavašič P., Pirkmajer S., Pavlin M. (2019). Comparison of the Effects of Metformin on MDA-MB-231 Breast Cancer Cells in a Monolayer Culture and in Tumor Spheroids as a Function of Nutrient Concentrations. Biochem. Biophys. Res. Commun..

[41] Minami F., Sasaki N., Shichi Y., Gomi F., Michishita M., Ohkusu-Tsukada K., Toyoda M., Takahashi K., Ishiwata T. (2021). Morphofunctional analysis of human pancreatic cancer cell lines in 2-and 3-dimensional cultures. Sci. Rep..

[42] Lauri C., Chiurchioni L., Russo V.M., Zannini L., Signore A. (2022). PSMA Expression in Solid Tumors beyond the Prostate Gland: Ready for Theranostic Applications?. J. Clin. Med..

[43] Oh J.M., Gangadaran P., Rajendran R.L., Hong C.M., Lee J., Ahn B.-C. (2022). Different Expression of Thyroid-Specific Proteins in Thyroid Cancer Cells between 2-Dimensional (2D) and 3-Dimensional (3D) Culture Environment. Cells.

[44] Chauhan G., Wang X., Yousry C., Gupta V. (2023). Scalable Production and In Vitro Efficacy of Inhaled Erlotinib Nanoemulsion for Enhanced Efficacy in Non-Small Cell Lung Cancer (NSCLC). Pharmaceutics.

[45] Lopez E., Kamboj S., Chen C., Wang Z., Kellouche S., Leroy-Dudal J., Carreiras F., Lambert A., Aimé C. (2023). In Vitro Models of Ovarian Cancer: Bridging the Gap between Pathophysiology and Mechanistic Models. Biomolecules.

[46] Fiegl H., Hagenbuchner J., Kyvelidou C., Seeber B., Sopper S., Tsibulak I., Wieser V., Reiser E., Roessler J., Huhtinen K. (2022). Dubious Effects of Methadone as an “Anticancer” Drug on Ovarian Cancer Cell-Lines and Patient-Derived Tumor-Spheroids. Gynecol. Oncol..

[47] Glaudemans A.W.J.M., de Vries E.F.J., Galli F., Dierckx R.A.J.O., Slart R.H.J.A., Signore A. (2013). The use of F-FDG-PET/CT for diagnosis and treatment monitoring of inflammatory and infectious diseases. Clin. Dev. Immunol..

[48] Amorim B.J., Schaarschmidt B.M., Grueneisen J., Tajmir S., Umutlu L., Signore A., Catalano O.A. (2020). Nuclear Medicine Imaging of Infection/Inflammation by PET/CT and PET/MR. Nuclear Medicine in Infectious Diseases.

[49] Anzola L.K., Glaudemans A.W.J.M., Dierckx R.A.J.O., Martinez F.A., Moreno S., Signore A. (2019). Somatostatin Receptor Imaging by SPECT and PET in Patients with Chronic Inflammatory Disorders: A Systematic Review. Eur. J. Nucl. Med. Mol. Imaging.

[50] Auletta S., Varani M., Horvat R., Galli F., Signore A., Hess S. (2019). PET Radiopharmaceuticals for Specific Bacteria Imaging: A Systematic Review. J. Clin. Med..

[51] Catalano O.A., Horn G.L., Signore A., Iannace C., Lepore M., Vangel M., Luongo A., Catalano M., Lehman C., Salvatore M. (2017). PET/MR in Invasive Ductal Breast Cancer: Correlation between Imaging Markers and Histological Phenotype. Br. J. Cancer.

[52] Signore A., Galli F., Auletta S., Briganti E., Lauri C. (2016). Molecular imaging of cancer microenvironment. Nucleus.

[53] Signore A., Bonfiglio R., Varani M., Galli F., Campagna G., Desco M., Cussó L., Mattei M., Wunder A., Borri F. (2023). Radioimmune Imaging of A4β7 Integrin and TNFα for Diagnostic and Therapeutic Applications in Inflammatory Bowel Disease. Pharmaceutics.

[54] Tornes A.J.K., Stenberg V.Y., Larsen R.H., Bruland Ø.S., Revheim M.-E., Juzeniene A. (2022). Targeted Alpha Therapy with the ^224^Ra/^212^Pb-TCMC-TP-3 Dual Alpha Solution in a Multicellular Tumor Spheroid Model of Osteosarcoma. Front. Med..

[55] Abramenkovs A., Hariri M., Spiegelberg D., Nilsson S., Stenerlöw B. (2022). Ra-223 Induces Clustered DNA Damage and Inhibits Cell Survival in Several Prostate Cancer Cell Lines. Transl. Oncol..

[56] Ingargiola M., Runge R., Heldt J.-M., Freudenberg R., Steinbach J., Cordes N., Baumann M., Kotzerke J., Brockhoff G., Kunz-Schughart L.A. (2014). Potential of a Cetuximab-Based Radioimmunotherapy Combined with External Irradiation Manifests in a 3-D Cell Assay. Int. J. Cancer.

[57] Song H., Shahverdi K., Huso D.L., Esaias C., Fox J., Liedy A., Zhang Z., Reilly R.T., Apostolidis C., Morgenstern A. (2008). 213Bi (α-Emitter)–Antibody Targeting of Breast Cancer Metastases in the Neu-N Transgenic Mouse Model. Cancer Res..

[58] Lundsten S., Spiegelberg D., Stenerlow B., Nestor M. (2019). The HSP90 Inhibitor Onalespib Potentiates 177Lu DOTATATE Therapy in Neuroendocrine Tumor Cells. Int. J. Oncol..

[59] Kasten B.B., Gangrade A., Kim H., Fan J., Ferrone S., Ferrone C.R., Zinn K.R., Buchsbaum D.J. (2018). ^212^Pb-Labeled B7-H3-Targeting Antibody for Pancreatic Cancer Therapy in Mouse Models. Nucl. Med. Biol..

[60] Akil H., Quintana M., Raymond J.H., Billoux T., Benboubker V., Besse S., Auzeloux P., Delmas V., Petit V., Larue L. (2021). Efficacy of Targeted Radionuclide Therapy Using [^131^I]ICF01012 in 3D Pigmented BRAF- and NRAS-Mutant Melanoma Models and In Vivo NRAS-Mutant Melanoma. Cancers.

[61] de Kruijff R.M., van der Meer A.J.G.M., Windmeijer C.A.A., Kouwenberg J.J.M., Morgenstern A., Bruchertseifer F., Sminia P., Denkova A.G. (2018). The Therapeutic Potential of Polymersomes Loaded with 225Ac Evaluated in 2D and 3D in Vitro Glioma Models. Eur. J. Pharm. Biopharm..

[62] Gaze M., Mairs R., Boyack S., Wheldon T., Barrett A. (1992). 131I-Meta-Iodobenzylguanidine Therapy in Neuroblastoma Spheroids of Different Sizes. Br. J. Cancer.

[63] Neshasteh-Riz A., Angerson W., Reeves J., Smith G., Rampling R., Mairs R. (1997). Incorporation of Iododeoxyuridine in Multicellular Glioma Spheroids: Implications for DNA-Targeted Radiotherapy Using Auger Electron Emitters. Br. J. Cancer.

[64] Lauri C., Campagna G., Aloisi F., Posa A., Iezzi R., Sirignano P., Taurino M., Signore A. (2023). How to Combine CTA, 99mTc-WBC SPECT/CT, and [^18^F]FDG PET/CT in Patients with Suspected Abdominal Vascular Endograft Infections?. Eur. J. Nucl. Med. Mol. Imaging.

[65] Prosperi D., Carideo L., Russo V., Meucci R., Campagna G., Lastoria S., Signore A. (2023). A Systematic Review on Combined [^18^F]FDG and 68Ga-SSA PET/CT in Pulmonary Carcinoid. J. Clin. Med..

[66] Lauri C., Signore A., Campagna G., Aloisi F., Taurino M., Sirignano P. (2023). [^18^F]FDG Uptake in Non-Infected Endovascular Grafts: A Retrospective Study. Diagnostics.

[67] Signore A., Lauri C., Bianchi M.P., Pelliccia S., Lenza A., Tetti S., Martini M.L., Franchi G., Trapasso F., De Biase L. (2022). [^18^F]FDG PET/CT in Patients Affected by SARS-CoV-2 and Lymphoproliferative Disorders and Treated with Tocilizumab. J. Pers. Med..

[68] Silveri G.G., Chiurchioni L., Magi L., Ambrosini V., Pizzichini P., Russo V., Rinzivillo M., Panzuto F., Signore A., Prosperi D. (2022). The impact of [^18^F]FDG PET/CT on clinical management in gastro-entero-pancreatic neuroendocrine tumors G1. Eur. J. Nucl. Med. Mol. Imaging.

[69] Magi L., Prosperi D., Lamberti G., Marasco M., Ambrosini V., Rinzivillo M., Campana D., Gentiloni G., Annibale B., Signore A. (2022). Role of [^18^F]FDG PET/CT in the Management of G1 Gastro-Entero-Pancreatic Neuroendocrine Tumors. Endocrine.

[70] Kelly C.J., Hussien K., Muschel R.J. (2012). 3D Tumour Spheroids as a Model to Assess the Suitability of [^18^F]FDG-PET as an Early Indicator of Response to PI3K Inhibition. Nucl. Med. Biol..

[71] Purohit N.K., Shah R.G., Adant S., Hoepfner M., Shah G.M., Beauregard J.-M. (2018). Potentiation of 177Lu-Octreotate Peptide Receptor Radionuclide Therapy of Human Neuroendocrine Tumor Cells by PARP Inhibitor. Oncotarget.

[72] Bentivoglio V., Varani M., Lauri C., Ranieri D., Signore A. (2022). Methods for Radiolabelling Nanoparticles: PET Use (Part 2). Biomolecules.

[73] Varani M., Bentivoglio V., Lauri C., Ranieri D., Signore A. (2022). Methods for Radiolabelling Nanoparticles: SPECT Use (Part 1). Biomolecules.

[74] Jahandar M., Zarrabi A., Shokrgozar M.A., Mousavi H. (2015). Synthesis, Characterization and Application of Polyglycerol Coated Fe_3_O_4_ Nanoparticles as a Nano-Theranostics Agent. Mater. Res. Express.

[75] Ünak P., Yasakçı V., Tutun E., Karatay K.B., Walczak R., Wawrowicz K., Żelechowska-Matysiak K., Majkowska-Pilip A., Bilewicz A. (2023). Multimodal Radiobioconjugates of Magnetic Nanoparticles Labeled with 44Sc and 47Sc for Theranostic Application. Pharmaceutics.

[76] Raghavan S., Mehta P., Ward M.R., Bregenzer M.E., Fleck E.M.A., Tan L., McLean K., Buckanovich R.J., Mehta G. (2017). Personalized Medicine–Based Approach to Model Patterns of Chemoresistance and Tumor Recurrence Using Ovarian Cancer Stem Cell Spheroids. Clin. Cancer Res..

[77] Jordan C.T., Guzman M.L., Noble M. (2006). Cancer Stem Cells. New Engl. J. Med..

[78] Páez D., Labonte M.J., Bohanes P., Zhang W., Benhanim L., Ning Y., Wakatsuki T., Loupakis F., Lenz H.-J. (2012). Cancer Dormancy: A Model of Early Dissemination and Late Cancer Recurrence. Clin. Cancer Res..

[79] Varzideh F., Mahmoudi E., Pahlavan S. (2019). Coculture with Noncardiac Cells Promoted Maturation of Human Stem Cell–Derived Cardiomyocyte Microtissues. J. Cell Biochem..

[80] Wessely A., Waltera A., Reichert T.E., Stöckl S., Grässel S., Bauer R.J. (2019). Induction of ALP and MMP9 Activity Facilitates Invasive Behavior in Heterogeneous Human BMSC and HNSCC 3D Spheroids. FASEB J..

[81] Zhang C., Yang Z., Dong D.L., Jang T.S., Knowles J.C., Kim H.W., Jin G.Z., Xuan Y. (2020). 3D culture technologies of cancer stem cells: Promising ex vivo tumor models. J. Tissue Eng..

[82] Zhang Z., Huang W., Lei M., He Y., Yan M., Zhang X., Zhao C. (2016). Laser-Triggered Intraocular Implant to Induce Photodynamic Therapy for Posterior Capsule Opacification Prevention. Int. J. Pharm..

[83] Sharma S. (2001). The Cost-Effectiveness of Photodynamic Therapy for Fellow Eyes with Subfoveal Choroidal Neovascularization Secondary to Age-Related Macular Degeneration. Ophthalmology.

[84] Karges J., Basu U., Blacque O., Chao H., Gasser G. (2019). Polymeric Encapsulation of Novel Homoleptic Bis(Dipyrrinato) Zinc(II) Complexes with Long Lifetimes for Applications as Photodynamic Therapy Photosensitisers. Angew. Chem. Int. Ed..

[85] Kumari P., Rompicharla S.V.K., Bhatt H., Ghosh B., Biswas S. (2019). Development of chlorin e6-conjugated poly (ethylene glycol)-poly (d, l-lactide) nanoparticles for photodynamic therapy. Nanomedicine.

[86] Di Giacomo A.M., Valente M., Cerase A., Lofiego M.F., Piazzini F., Calabrò L., Gambale E., Covre A., Maio M. (2019). Immunotherapy of Brain Metastases: Breaking a “Dogma”. J. Exp. Clin. Cancer Res..

[87] Herter S., Morra L., Schlenker R., Sulcova J., Fahrni L., Waldhauer I., Lehmann S., Reisländer T., Agarkova I., Kelm J.M. (2017). A novel three-dimensional heterotypic spheroid model for the assessment of the activity of cancer immunotherapy agents. Cancer Immunol. Immunother..

[88] Gupta N., Liu J.R., Patel B., Solomon D.E., Vaidya B., Gupta V. (2016). Microfluidics-based 3D Cell Culture Models: Utility in Novel Drug Discovery and Delivery Research. Bioeng. Transl. Med..

[89] Sun T., Zhang Y.S., Pang B., Hyun D.C., Yang M., Xia Y. (2021). Engineered Nanoparticles for Drug Delivery in Cancer Therapy. Angew. Chem. Int. Ed..

[90] Charron D.M., Chen J., Zheng G. (2015). Theranostic Lipid Nanoparticles for Cancer Medicine. Nanotechnol. Based Precis. Tools Detect. Treat. Cancer.

[91] Bao G., Mitragotri S., Tong S. (2013). Multifunctional Nanoparticles for Drug Delivery and Molecular Imaging. Annu. Rev. Biomed. Eng..

[92] Khan I., Khan M., Umar M.N., Oh D. (2015). Nanobiotechnology and Its Applications in Drug Delivery System: A Review. IET Nanobiotechnol..

[93] Gupta N., Al-Saikhan F.I., Patel B., Rashid J., Ahsan F. (2015). Fasudil and SOD Packaged in Peptide-Studded-Liposomes: Properties, Pharmacokinetics and Ex-Vivo Targeting to Isolated Perfused Rat Lungs. Int. J. Pharm..

[94] Figarol A., Gibot L., Golzio M., Lonetti B., Mingotaud A.-F., Rols M.-P. (2018). A Journey from the Endothelium to the Tumor Tissue: Distinct Behavior between PEO-PCL Micelles and Polymersomes Nanocarriers. Drug Deliv..

[95] Ahluwalia A., Jones M.K., Szabo S., Tarnawski A.S. (2013). Aberrant, Ectopic Expression of VEGF and VEGF Receptors 1 and 2 in Malignant Colonic Epithelial Cells. Implications for These Cells Growth via an Autocrine Mechanism. Biochem. Biophys. Res. Commun..

[96] Alexis F., Pridgen E., Molnar L.K., Farokhzad O.C. (2008). Factors Affecting the Clearance and Biodistribution of Polymeric Nanoparticles. Mol. Pharm..

[97] Alibolandi M., Alabdollah F., Sadeghi F., Mohammadi M., Abnous K., Ramezani M., Hadizadeh F. (2016). Dextran-b-Poly(Lactide-Co-Glycolide) Polymersome for Oral Delivery of Insulin: In Vitro and in Vivo Evaluation. J. Control. Release.

[98] Shao C., Chi J., Zhang H., Fan Q., Zhao Y., Ye F. (2020). Development of Cell Spheroids by Advanced Technologies. Adv. Mater. Technol..

[99] Millard M., Yakavets I., Zorin V., Kulmukhamedova A., Marchal S., Bezdetnaya L. (2017). Drug Delivery to Solid Tumors: The Predictive Value of the Multicellular Tumor Spheroid Model for Nanomedicine Screening. Int. J. Nanomed..

[100] Mehta G., Hsiao A.Y., Ingram M., Luker G.D., Takayama S. (2012). Opportunities and Challenges for Use of Tumor Spheroids as Models to Test Drug Delivery and Efficacy. J. Control. Release.

[101] Hirschhaeuser F., Walenta S., Mueller-Klieser W. (2010). Efficacy of Catumaxomab in Tumor Spheroid Killing Is Mediated by Its Trifunctional Mode of Action. Cancer Immunol. Immunother..

[102] Patra B., Peng C.-C., Liao W.-H., Lee C.-H., Tung Y.-C. (2016). Drug Testing and Flow Cytometry Analysis on a Large Number of Uniform Sized Tumor Spheroids Using a Microfluidic Device. Sci. Rep..

[103] Edmondson R., Broglie J.J., Adcock A.F., Yang L. (2014). Three-Dimensional Cell Culture Systems and Their Applications in Drug Discovery and Cell-Based Biosensors. Assay Drug Dev. Technol..

[104] Solomon M.A., Lemera J., D’Souza G.G.M. (2016). Development of an in Vitro Tumor Spheroid Culture Model Amenable to High-Throughput Testing of Potential Anticancer Nanotherapeutics. J. Liposome Res..

[105] Huo S., Ma H., Huang K., Liu J., Wei T., Jin S., Zhang J., He S., Liang X.-J. (2013). Superior Penetration and Retention Behavior of 50 Nm Gold Nanoparticles in Tumors. Cancer Res..

[106] Cagel M., Grotz E., Bernabeu E., Moretton M.A., Chiappetta D.A. (2017). Doxorubicin: Nanotechnological Overviews from Bench to Bedside. Drug Discov. Today.

[107] Sarisozen C., Abouzeid A.H., Torchilin V.P. (2014). The Effect of Co-Delivery of Paclitaxel and Curcumin by Transferrin-Targeted PEG-PE-Based Mixed Micelles on Resistant Ovarian Cancer in 3-D Spheroids and in Vivo Tumors. Eur. J. Pharm. Biopharm..

[108] Sarisozen C., Dhokai S., Tsikudo E.G., Luther E., Rachman I.M., Torchilin V.P. (2016). Nanomedicine Based Curcumin and Doxorubicin Combination Treatment of Glioblastoma with ScFv-Targeted Micelles: In Vitro Evaluation on 2D and 3D Tumor Models. Eur. J. Pharm. Biopharm..

[109] Wang X., Tang H., Wang C., Zhang J., Wu W., Jiang X. (2016). Phenylboronic Acid-Mediated Tumor Targeting of Chitosan Nanoparticles. Theranostics.

[110] Zong T., Mei L., Gao H., Cai W., Zhu P., Shi K., Chen J., Wang Y., Gao F., He Q. (2014). Synergistic Dual-Ligand Doxorubicin Liposomes Improve Targeting and Therapeutic Efficacy of Brain Glioma in Animals. Mol. Pharm..

[111] Kim T.-H., Mount C.W., Gombotz W.R., Pun S.H. (2010). The Delivery of Doxorubicin to 3-D Multicellular Spheroids and Tumors in a Murine Xenograft Model Using Tumor-Penetrating Triblock Polymeric Micelles. Biomaterials.

[112] Saluja V., Mishra Y., Mishra V., Giri N., Nayak P. (2021). Dendrimers Based Cancer Nanotheranostics: An Overview. Int. J. Pharm..

[113] Mishra V., Singh M., Nayak P. (2021). Smart Functionalised-Dendrimeric Medicine in Cancer Therapy. Dendrimers in Nanomedicine.

[114] Mishra V., Yadav N., Saraogi G.K., Tambuwala M.M., Giri N. (2019). Dendrimer Based Nanoarchitectures in Diabetes Management: An Overview. Curr. Pharm. Des..

[115] Mishra V., Singh M., Mishra Y., Charbe N., Nayak P., Sudhakar K., Aljabali A.A.A., Shahcheraghi S.H., Bakshi H., Serrano-Aroca Á. (2021). Nanoarchitectures in Management of Fungal Diseases: An Overview. Appl. Sci..

[116] Jain N.K., Tare M.S., Mishra V., Tripathi P.K. (2015). The Development, Characterization and in Vivo Anti-Ovarian Cancer Activity of Poly(Propylene Imine) (PPI)-Antibody Conjugates Containing Encapsulated Paclitaxel. Nanomedicine.

[117] Suttee A., Singh G., Yadav N., Pratap Barnwal R., Singla N., Prabhu K.S., Mishra V. (2019). A Review on Status of Nanotechnology in Pharmaceutical Sciences. Int. J. Drug Deliv. Technol..

[118] Rompicharla S.V.K., Kumari P., Bhatt H., Ghosh B., Biswas S. (2019). Biotin Functionalized PEGylated Poly(Amidoamine) Dendrimer Conjugate for Active Targeting of Paclitaxel in Cancer. Int. J. Pharm..

[119] Riyaz B., Sudhakar K., Mishra V. (2019). Quantum Dot-Based Drug Delivery for Lung Cancer. Nanotechnology-Based Targeted Drug Delivery Systems for Lung Cancer.

[120] Mishra V., Gurnany E., Mansoori M.H. (2017). Quantum Dots in Targeted Delivery of Bioactives and Imaging. Nanotechnology-Based Approaches for Targeting and Delivery of Drugs and Genes.

[121] Mangeolle T., Yakavets I., Lequeux N., Pons T., Bezdetnaya L., Marchal F. (2019). The Targeting Ability of Fluorescent Quantum Dots to the Folate Receptor Rich Tumors. Photodiagnosis Photodyn. Ther..

[122] Singh M., Nayak P., Mishra V. (2020). Carbon Nanotubes in Treatment of Arthritis: An Overview. Eur. J. Mol. Clin. Med..

[123] Mishra V., Singh M., Nayak P., Sriram P., Suttee A. (2020). Carbon Nanotubes as Emerging Nanocarriers in Drug Delivery: An Overview. Int. J. Pharm. Qual..

[124] Mishra V., Kesharwani P., Jain N.K. (2018). Biomedical Applications and Toxicological Aspects of Functionalized Carbon Nanotubes. Crit. Rev. Ther. Drug Carr. Syst..

[125] Kesharwani P., Mishra V., Jain N.K. (2015). Validating the Anticancer Potential of Carbon Nanotube-Based Therapeutics through Cell Line Testing. Drug Discov. Today.

[126] Kabadi P.K., Rodd A.L., Simmons A.E., Messier N.J., Hurt R.H., Kane A.B. (2019). A Novel Human 3D Lung Microtissue Model for Nanoparticle-Induced Cell-Matrix Alterations. Part Fibre. Toxicol..

[127] Pandey H., Rani R., Agarwal V. (2016). Liposome and Their Applications in Cancer Therapy. Braz. Arch. Biol. Technol..

[128] Rodallec A., Sicard G., Giacometti S., Carré M., Pourroy B., Bouquet F., Savina A., Lacarelle B., Ciccolini J., Fanciullino R. (2018). From 3D Spheroids to Tumor Bearing Mice: Efficacy and Distribution Studies of Trastuzumab-Docetaxel Immunoliposome in Breast Cancer. Int. J. Nanomed..

[129] Kaur J., Mishra V., Singh S.K., Gulati M., Kapoor B., Chellappan D.K., Gupta G., Dureja H., Anand K., Dua K. (2021). Harnessing Amphiphilic Polymeric Micelles for Diagnostic and Therapeutic Applications: Breakthroughs and Bottlenecks. J. Control. Release.

[130] Kedar U., Phutane P., Shidhaye S., Kadam V. (2010). Advances in Polymeric Micelles for Drug Delivery and Tumor Targeting. Nanomedicine.

[131] Patil A., Mishra V., Thakur S., Riyaz B., Kaur A., Khursheed R., Patil K., Sathe B. (2018). Nanotechnology Derived Nanotools in Biomedical Perspectives: An Update. Curr. Nanosci..

[132] Kumari P., Jain S., Ghosh B., Zorin V., Biswas S. (2017). Polylactide-Based Block Copolymeric Micelles Loaded with Chlorin E6 for Photodynamic Therapy: In Vitro Evaluation in Monolayer and 3D Spheroid Models. Mol. Pharm..

[133] Mishra V., Nayak P., Singh M., Tambuwala M.M., Aljabali A.A., Chellappan D.K., Dua K. (2021). Pharmaceutical Aspects of Green Synthesized Silver Nanoparticles: A Boon to Cancer Treatment. Anticancer Agents Med. Chem..

[134] Arora N., Shome R., Ghosh S.S. (2019). Deciphering Therapeutic Potential of PEGylated Recombinant PTEN-Silver Nanoclusters Ensemble on 3D Spheroids. Mol. Biol. Rep..

[135] Kaur M., Sudhakar K., Mishra V. (2019). Fabrication and Biomedical Potential of Nanogels: An Overview. Int. J. Polym. Mater. Polym. Biomater..

[136] Saraogi G.K., Tholiya S., Mishra Y., Mishra V., Albutti A., Nayak P., Tambuwala M.M. (2022). Formulation Development and Evaluation of Pravastatin-Loaded Nanogel for Hyperlipidemia Management. Gels.

[137] Cheng X., Zeng X., Li D., Wang X., Sun M., He L., Tang R. (2019). TPGS-Grafted and Acid-Responsive Soy Protein Nanogels for Efficient Intracellular Drug Release, Accumulation, Penetration in 3D Tumor Spheroids of Drug-Resistant Cancer Cells. Mater. Sci. Eng. C.

[138] Gupta C., Prakash D., Gupta S. (2017). Cancer treatment with nanodiamonds. Front. Biosci..

[139] Madamsetty V.S., Sharma A., Toma M., Samaniego S., Gallud A., Wang E., Pal K., Mukhopadhyay D., Fadeel B. (2019). Tumor Selective Uptake of Drug-Nanodiamond Complexes Improves Therapeutic Outcome in Pancreatic Cancer. Nanomedicine.

[140] Yao Q., Choi J.H., Dai Z., Wang J., Kim D., Tang X., Zhu L. (2017). Improving Tumor Specificity and Anticancer Activity of Dasatinib by Dual-Targeted Polymeric Micelles. ACS Appl. Mater. Interfaces.

[141] Varani M., Bentivoglio V., Serafinelli M., Lauri C., Signore A.A. (2022). Designed radiolabelled Poly (Lactic-co-Glycolic Acid) nanoparticle for theragnostic applications. Eur. J. Nucl. Med. Mol. Imaging.

[142] Varani M., Galli F., Capriotti G., Mattei M., Cicconi R., Campagna G., Panzuto F., Signore A. (2020). Theranostic Designed Near-Infrared Fluorescent Poly (Lactic-Co-Glycolic Acid) Nanoparticles and Preliminary Studies with Functionalized VEGF-Nanoparticles. J. Clin. Med..

[143] Varani M., Galli F., Bentivoglio V., Signore A., Signore A. (2022). Particles and nanoparticles in nuclear medicine: Basic principles and instrumentation. Nuclear Medicine and Molecular Imaging.

[144] Varani M., Campagna G., Bentivoglio V., Serafinelli M., Martini M.L., Galli F., Signore A. (2021). Synthesis and Biodistribution of 99mTc-Labeled PLGA Nanoparticles by Microfluidic Technique. Pharmaceutics.

[145] Bentivoglio V., Nayak P., Varani M., Lauri C., Signore A. (2023). Methods for Radiolabeling Nanoparticles (Part 3): Therapeutic Use. Biomolecules.

[146] Le T.T.D., Pham T.H., Nguyen T.N., Ngo T.H.G., Hoang T.M.N., Le Q.H. (2016). Evaluation of Anti-HER2 ScFv-Conjugated PLGA–PEG Nanoparticles on 3D Tumor Spheroids of BT474 and HCT116 Cancer Cells. Adv. Nat. Sci. Nanosci. Nanotechnol..

[147] Zhang X., Chen X., Zhao Y. (2022). Nanozymes: Versatile platforms for cancer diagnosis and therapy. Nano-Micro Lett..

[148] Carvalho S.M., Mansur A.A., da Silveira I.B., Pires T.F., Victória H.F., Krambrock K., Leite M.F., Mansur H.S. (2023). Nanozymes with Peroxidase-like Activity for Ferroptosis-Driven Biocatalytic Nanotherapeutics of Glioblastoma Cancer: 2D and 3D Spheroids Models. Pharmaceutics.

